# Integrated travel network model for studying epidemics: Interplay between journeys and epidemic

**DOI:** 10.1038/srep11401

**Published:** 2015-06-15

**Authors:** Zhongyuan Ruan, Chaoqing Wang, Pak Ming Hui, Zonghua Liu

**Affiliations:** 1 Department of Physics, East China Normal University, Shanghai, 200062, China; 2 Center for Network Science, Central European University; 3 Department of Physics, The Chinese University of Hong Kong, Shatin, New Territories, Hong Kong

## Abstract

The ease of travelling between cities has contributed much to globalization. Yet, it poses a threat on epidemic outbreaks. It is of great importance for network science and health control to understand the impact of frequent journeys on epidemics. We stress that a new framework of modelling that takes a traveller’s viewpoint is needed. Such integrated travel network (ITN) model should incorporate the diversity among links as dictated by the distances between cities and different speeds of different modes of transportation, diversity among nodes as dictated by the population and the ease of travelling due to infrastructures and economic development of a city, and round-trip journeys to targeted destinations via the paths of shortest travel times typical of human journeys. An example is constructed for 116 cities in China with populations over one million that are connected by high-speed train services and highways. Epidemic spread on the constructed network is studied. It is revealed both numerically and theoretically that the traveling speed and frequency are important factors of epidemic spreading. Depending on the infection rate, increasing the traveling speed would result in either an enhanced or suppressed epidemic, while increasing the traveling frequency enhances the epidemic spreading.

Controlling an epidemic, e.g. severe acute respiratory syndrome (SARS), H1N1 swine influenza, and Ebola, in the midst of frequent movements of infected persons via cars, trains, and aeroplanes poses a challenging problem. In network science, much effort and progress has been made on understanding epidemics in single-layered networks^1^,^2^,^3^,^4^,^5^,^6^,^7^,^8^,^9^,^10^,^11^,^12^,^13^,^14^,^15^,^16^,^17^,^18^,^19^,^20^ and multi-layered networks^21^,^22^,^23^,^24^,^25^,^26^,^27^,^28^. In single-layered static networks with an immobile agent at each node, for example, no finite epidemic threshold exists for scale-free (SF) networks and a tiny initial infection eventually spreads^11^. A delicate balance between the number of high degree nodes and the topological distance between them^29^ is shown to be crucial. The same result holds for reaction-diffusion models with random diffusion of agents among nodes with infections only among the agents momentarily on the same node^6^. Recently, how human dynamics affects an epidemic has become the focus of research^14^,^18^,^30^,^31^,^32^,^33^, but the diversity of links and the time spending on journeys are largely ignored. Real-life networks, e.g. power grids and the internet, are often multi-layered networks^34^,^35^, with their mutual influence and cascades being hot research topics^36^,^37^. Epidemics in two-layered networks also received much attention^21^,^22^,^23^,^24^,^25^,^26^, and the layer for infection processes actually shares the same set of nodes with the layer for information exchanges.

For diseases spreading through human contacts, it is most important to understand the impact of frequent journeys. There exist many single and multi-layered transportation network models^38^,^39^,^40^,^41^,^42^,^43^, with the layers representing networks of airports, railways, highways, etc. coupled together. To incorporate epidemics, however, random diffusion of people on such networks will be an oversimplification, as a journey involves a planned route to a destination using mixed modes of transportation. These directed movements should be incorporated in studying epidemics.

The ease and speed of inter-city travels offered by the growth in the airline and high-speed train^44^ industries and better highways has contributed to making our Earth a global village. These inter-city travels readily spread a disease to different places. However, the big populations in major cities and densely packed travellers on multiple means of transportation of various speeds add further complications. A reliable framework for studying the effects of travelling on epidemics has yet to be constructed. Earlier works on epidemics in airport and railway networks often modelled journeys as random diffusion of agents^4^,^5^,^45^. The obvious shortcomings are: (i) real journeys typically involve multiple means of transportation instead of agents all travelling the same way; (ii) neighboring stations have different distances that affect the chance of infection instead of identical distance between adjacent nodes; (iii) real journeys are round-trip with an destination instead of random diffusion. It should be noted that intra-city travel is also inhomogeneous. It is, therefore, of fundamental importance to construct a framework incorporating the differences in travelling means and distances between cities. We propose here such a framework to incorporate inhomogeneity among the links and round-trip journeys with intended destination. It is found that infections at the links greatly affect the epidemic threshold, and the traveling speed and frequency are key factors in determining the extent of an epidemic.

## Results

### An integrated travel network (ITN) model

**Figure 1:**
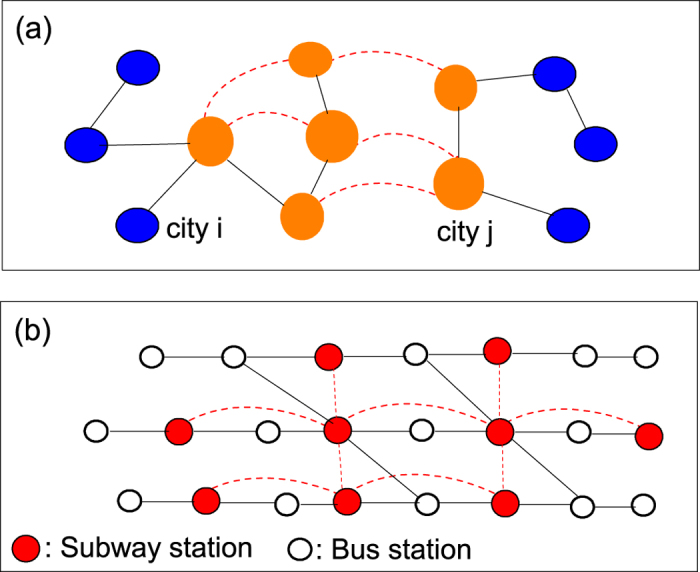
Schematic illustration of transportation networks. (**a**) Schematic inter-city transportation network illustrating the inhomogeneity in the links, e.g. dashed lines for higher speed transportation such as a part of an airport network or high-speed railway network and other cities (in blue) are connected through a part of the highway network. (**b**) Schematic intra-city transportation illustrating the link inhomogeneity, e.g. nodes (filled) connected by subways (dashed lines) and other nodes (open) connected by bus routes (solid lines).

Our integrated travel network (ITN) model accounts for different means of transportation by different kinds of links. Figure 1(a) shows schematically an inter-city transportation network emphasizing its link inhomogeneity: Links of faster transportation (dashed lines), e.g. airlines and high-speed trains, connecting major cities and links of slower transportation (solid lines), e.g. highways, connecting to surrounding cities (blue nodes) via part of a highway network.

**Figure 2:**
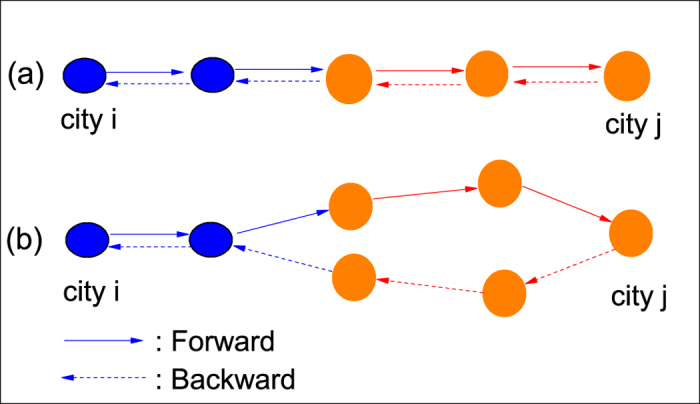
Round-trip journey with targeted destination. (**a**) Agent could follow the same path back or (**b**) take an alternative path back. We take the path of the shortest travel time.

A journey starts from a city *i* to an intended destination *j* through intermediate places along the path that takes the shortest time, which necessarily invoke the actual distance between two cities and the mode of transportation. The return journey could follow the same path or an alternative path, as depicted in Fig. 2(a,b). The ITN aims to incorporate the key features of how human travel, namely round-trip journeys of shortest time through multiple means of transportation. Here, we invoke the *travel time*, which depends on the distance and the means of transportation, as the key factor, instead of the effective distance^43^. Instead of emphasizing the multi-layered network structure as in previous works, ITN takes a traveller’s viewpoint that journeys take place in a single-layered undetachable network with a diversity of links connecting cities representing an inhomogeneous transportation network, see Methods for details. It aims to provide a step closer to a realistic description of human journeys and an alternative platform for studying epidemics on which finer and further details on local area transportation could be added.

### Epidemic spreading on ITN

**Figure 3:**
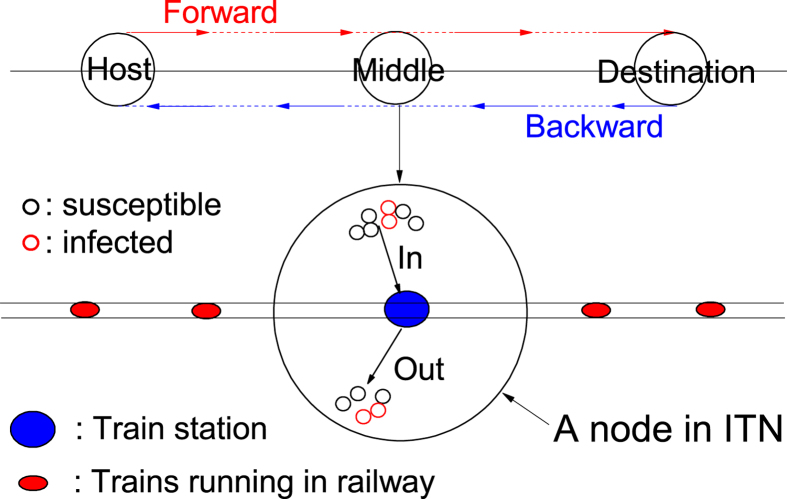
Schematic illustration of the key points in ITN. An agent starts a round-trip journey from his home city (Host) via the path of shortest travel time (Forward path) towards the destination via many other cities (Middle) along the path. After remaining at the destination for some time steps, he takes a return trip (Backward) back home. Other agents may join or leave. A link is divided into segments (red circles) according to the travel time between stations. During a journey, an agent would encounter passengers who are infected (red open circles) or susceptible to an infection (black open circles).

Contacts during journeys are important for epidemics. An example is the 2009 H1N1 cases in a Singapore’s hospital that 116 of 152 patients in two months were classified as air travel-associated imported cases^46^. The time that travellers meet becomes a crucial factor. It is related to the length of a link and how fast agents travel on it. As a minimum model, we consider two speeds *v*_*s*_ and *v*_*f*_ with *v*_*s*_ < *v*_*f*_ (see solid and dashed lines in Fig. 1) representing slower and faster transportation. An agent starts a round-trip journey from a node (home) to a destination chosen randomly (upper Fig. 3) through intermediate (middle) nodes along the path of shortest travel time^18^. Let *r*_*ij*_ be the distance between neighbouring nodes *i* and *j*. The time travelling on the link is


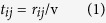


with *v* = *v*_*s*_ or *v*_*f*_ depending on the type of transportation. To account for travel time, a link from node *i* to node *j* is divided into *τ*_*ij*_ segments, with *τ*_*ij*_ = *t*_*ij*_ if *mod*(*r*_*ij*_,*v*) = 0 and *τ*_*ij*_ = *int*(*t*_*ij*_) + 1 if *mod*(*r*_*ij*_,*v*) ≠ 0 (lower Fig. 3), where *mod*(*x,y*) represents the modulo operation and *int*(*x*) taking the integral part of *x*.

For epidemic on ITN, we invoke the susceptible-infected-susceptible (SIS) model^6^,^9^,^10^,^11^,^12^,^13^,^14^,^15^,^16^,^17^. A susceptible agent will be infected if it contacts an infected agent, with an infectious rate *β*. There are travelling and non-travelling agents in a population. Generally, people travelling are in closer contact and have a higher infectious rate *β*_2_ than the non-travelling agents with *β*_1_^47^. An infected agent recovers and becomes susceptible with a recovery rate *μ*. For travelling agents, we assume that infections take place only among agents in the same segment *k*_*r*_ (1 ≤ *k*_*r*_ ≤ *τ*_*ij*_) of a link. For non-travelling agents, the SIS process is confined to non-travelling agents at the same node. Explicitly, a non-travelling susceptible agent at node *i* has a probability 1−(1−*β*_1_)^*n*^_*i,I*_ to be infected at a time step, when there are *n*_*i,I*_ infected non-travelling agents at the node. Similarly, a susceptible agent at a segment of a link has a probability 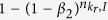 to be infected when there are 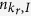 infected agents at that section *k_r_*.

### An example of ITN: China’s big city network

**Figure 4:**
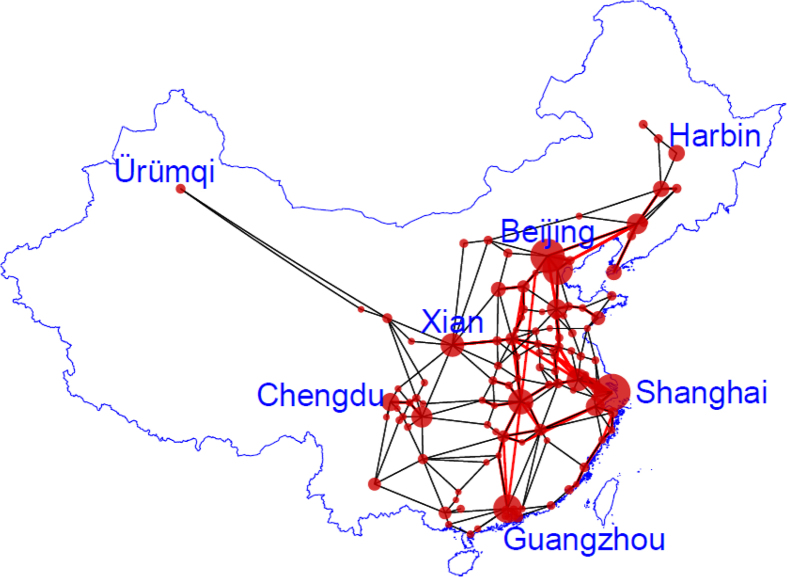
An example of ITN. An integrated travel network (ITN) constructed based on high-speed railway services (red links) and highway network (black lines) in China for 116 cities with a population larger than one million. The cities are represented by nodes of different sizes according to the populations. This figure was generated by R.

Buses on highways and high-speed trains in China together provide an example of ITN. To include a large population and to reduce the number of nodes, we consider 116 cities with population over one million (see Table S1 in Supplementary Information (SI)). From high-speed train schedule, 61 cities are served by routes of high-speed trains. For the remaining 55 cities, we construct the highway links as follows. A highway link is added between two cities in the same province or two neighbouring provinces when there is a highway between them. Finally, highway links are added to connect neighboring highway and high-speed railway nodes in the same province. Figure 4 shows the resulting ITN of 116 cities with two types of links. We give the structural properties in SI. It has a mean degree 〈*k*〉 = 4.25 and a high clustering coefficient of *C* = 0.35. The degree distribution is shown in Fig. S1(a) in SI. Table S2 in SI gives the lengths of the links.

Typically, travels between major cities and/or nearby cities are more frequent. This was modelled by assigning weights 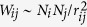 to a link, where *N*_*i*_ denotes the population at node *i* and *r*_*ij*_ the distance between nodes *i* and *j*^48^,^49^. To incorporate factors including transportation infrastructure and convenience, we modified the weight in ITN to


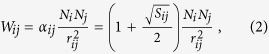


where *S*_*ij*_ represents the daily services of high-speed trains between nodes *i* and *j* and thus an indication of how convenient it is, and *S*_*ij*_ = 0 for highway links. Values of *S*_*ij*_ as obtained by train schedules are listed in Table S2 in SI. Summing *W*_*ij*_ for the *k*_*i*_ links give the weight *W*_*i*_ of node *i* as


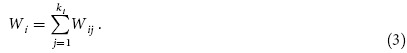


To set up a model for simulations, we measure population in units of 5000 and distance *r*_*ij*_ in kilometers. Thus cities of *N*_*i*_ ≥ 200 are considered and *N*_*i*_ is 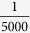 of the real population. The corresponding weight distribution is shown in Fig. S1(b) in SI. Sensitivity to the choice of measuring populations in lots of 5000 is tested in Fig. S2 in SI. In each time step, 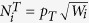 agents starts a round-trip journey from node *i*, where the parameter *p*_*T*_ is chosen so that 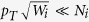, i.e., people travelling are fewer than a city’s residents. It is related to the small fraction *f* of the total population 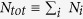 starting a journey every time step by


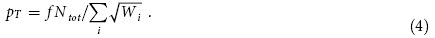


An agent from node *i* picks a destination *j* according to the probability


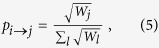


and follows the path of shortest travel time. An agent typically travels on slower transportation in the local area before transferring to high-speed train followed by local transportation to the destination. ITN captures the inhomogeneous means of travelling better than multi-layered networks. An agent spends some time at the destination before the return trip begins, which is taken to be 5 time steps corresponding to 5 hours^50^,^51^. Returning to home city, an agent becomes a non-traveller until the next journey. Figure 3 shows a schematic journey. The travelling dynamics leads to a steady state in which 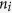 residents among 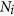 are non-travellers at node *i*. The number of all non-travellers 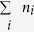 depends on *f* (see Fig. S2 in SI) linearly for *f* ≤ 0.01. We thus take *f* = 0.01. The values of *n*_*i*_ and 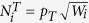 for the 116 cities are shown in Fig. S3 in SI.

### Epidemic spreading on China’s ITN network

**Figure 5:**
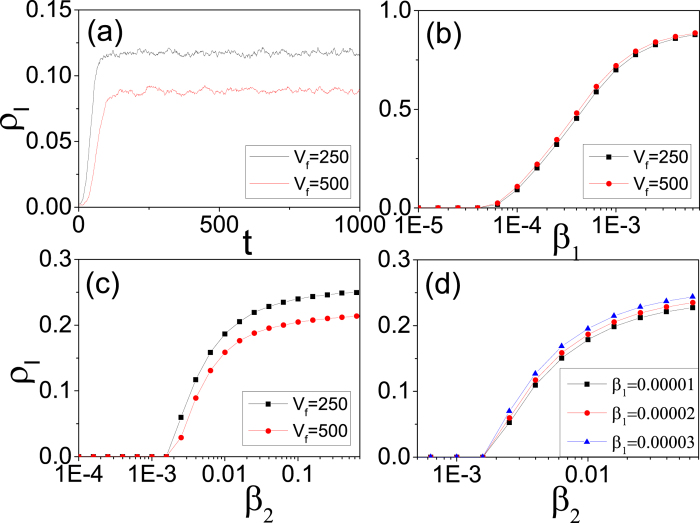
Effect of different parameters on infected density. (**a**) Time evolution of *ρ*_*I*_ with *β*_1_ = 2 × 10^−5^ and *β*_2_ = 0.004, for two values of train speed *v*_*f*_ = 250 and 500. (**b**) *ρ*_*I*_ as a function of the parameter *β*_1_ with *β*_1_ = *β*_2_, for two values of train speed *v*_*f*_ = 250 (squares) and 500 (dots). (**c**) *ρ*_*I*_ as a function of the parameter *β*_2_ with *β*_1_ = 2 × 10^−5^ < *β*_1*c*_ ≈ 4 × 10^−5^, for two values of train speed *v*_*f*_ = 250 and 500. (**d**) *ρ*_*I*_ as a function of the parameter *β*_2_ with *v*_*f*_ = 250, for three different values of *β*_1_ = 1 × 10^−5^ (squares), 2 × 10^−5^ (dots) and 3 × 10^−5^ (triangles).

Let *v*_*s*_ = 100 (km/h) be the highway traffic speed and *v*_*f*_* *> *v*_*s*_ be speed of high-speed train. The speeds and *r*_*ij*_ determine the time *τ*_*ij*_ of each link. After the travelling population reaches the steady state, the SIS process is initialized by assigning 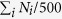 agents randomly as infected at *t* = 0. Practically, uniformly distributed initial infection speeds up the approach to the steady state. The recovery rate is fixed at *μ* = 0.1. Let *ρ*_*I*_ be the fraction of infected agents. Figure 5(a) shows *ρ*_*I*_(*t*) for *β*_1_ = 2 × 10^−5^ and *β*_2_ = 0.004, for two values of *v*_*f*_ = 250 and 500. An epidemic steady state is reached quickly. As a higher 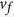 shortens the time on the links that the infection rate is higher, *ρ*_*I*_ is smaller for higher *v*_*f*_. Figure 5(b) shows the steady state *ρ*_*I*_ for *β*_1_ = *β*_2_. There exists a threshold *β*_1*c*_ ≈ 4 × 10^−5^ above which *ρ*_*I*_ ≠ 0.

As *β*_2_* *> *β*_1_ generally, Fig. 5(c) shows *ρ*_*I*_ (*β*_2_) after setting *β*_1_ = 2 × 10^−5^ < *β*_1*c*_, for two values of *v_f_*. Figure 5(d) shows *ρ*_*I*_(*β*_2_) for three different values of *β*_1_ < *β*_1*c*_. It is found that *β*_2*c*_ remains unchanged for different *β*_1_ < *β*_1*c*_. It is reasonable in that when the outbreaks come from infections in journeys, the infection rate *β*_1_ of non-travellers is irrelevant to the threshold *β*_2*c*_. However, for *β*_2_* *> *β*_2*c*_, a higher *β*_1_ leads to a higher *ρ*_*I*_.

**Figure 6:**
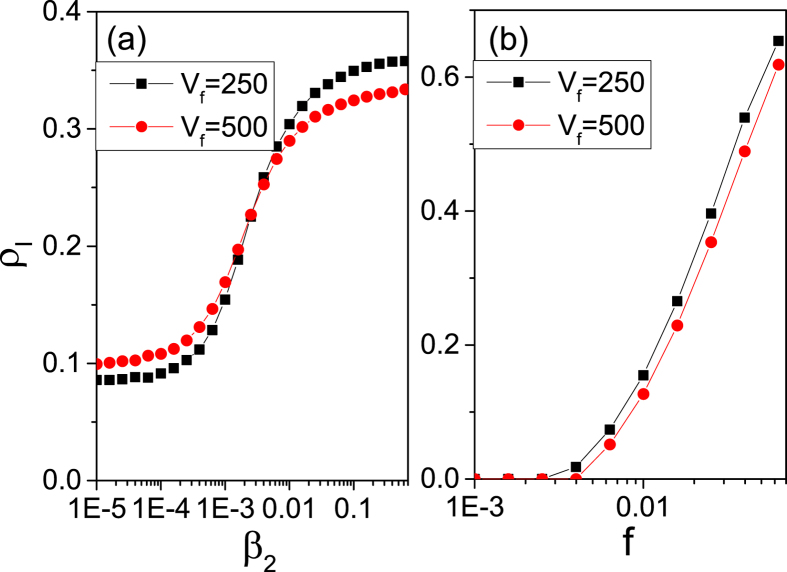
Effect of different parameters on infected density. (**a**) 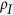 as a function of the parameter *β*_2_ with *β*_1_ = 10^−4^* *> *β*_1*c*_, for two values of train speed *v*_*f*_ = 250 (squares) and 500 (dots). (**b**) *ρ*_*I*_ as a function of *f* with *β*_1_ = 2 × 10^−5^ < *β*_1*c*_ and *β*_2_ = 0.006* *> *β*_2*c*_, for *v*_*f*_ = 250 (squares) and 500 (dots).

Next, we set *β*_1_ = 10^−4^* *> *β*_1*c*_ and Fig. 6(a) shows that *ρ*_*I*_(*β*_2_) increases monotonically with *β*_2_, for *v*_*f*_ = 250 and 500. Here, *ρ*_*I*_ ≠ 0 for all *β*_2_. There exists a value *β*_2*c*′_ (*β*_2*c*′_ = 0.0025 for the case in Fig. 6(a) below (above) which *ρ*_*I*_ for *v*_*f*_ = 250 is lower (higher) than that for *v*_*f*_ = 500.

To summarize the findings in a physical picture, for *β*_2_  < *β*_2*c*′_, infections among non-travellers at the nodes dominate the epidemic process. A higher *v*_*f*_ (e.g. *v*_*f*_ = 500) reduces the time that agents spent on journeys and thus promotes infection. For *β*_2_  > *β*_2*c*′_, infections among travellers on journeys dominate the epidemic process. A higher *v*_*f*_ shortens the journey and suppresses infection.

For *β*_1_ = 2 × 10^−5^ < *β*_1*c*_ and *β*_2_ = 0.006* *> *β*_2*c*_, infections during journeys dominate. Figure 6(b) shows that *ρ*_*I*_ increases monotonically with the fraction of travellers *f*, with *ρ*_*I*_ for *v*_*f*_ = 500 smaller than that for *v*_*f*_ = 250 due to the shorter journey time.

## Discussion

We stressed the necessity of establishing a new framework for modelling journeys in modern times and their effects on epidemics. We illustrated the key ideas by presenting an integrated travel network constructed by considering geographic data, population data and transportation infrastructures in China. An example using only the high-speed trains and highways among the 116 cities of over a million population suffices for stressing the points. An ITN should include: (i) diversity among the links due to different distances and different speeds of transportation; (ii) diversity among the cities due to different population sizes, and transportation services often reflecting their economic growth; (iii) round-trip journeys to targeted destination via paths of shortest time; and (iv) different infection rates for travellers and non-travellers. The ITN can readily be extended to include details on local area transportation, multiple means of transportation, and journeys among different countries. For example, Fig. 1(b) shows schematically a local transportation network with stations (nodes) served by a subway network (dashed lines) and a bus network (solid lines). A journey includes generally travelling in both Fig. 1(a,b). Effects such as traffic congestion naturally emerge. As far as epidemics are concerned, faster and more convenient inter-city journeys would reduce the travel time during which passengers are crowded and thus suppress the chance of being infected, but they would also induce people to make more journeys and to farther places and thus spread a diseases more readily. Our ITN would serve as a good starting point for exploring the interplay of travelling and infection dynamics for many further work.

## Methods

### Degree and weight distributions of ITN

Highway buses and high-speed trains are the major means of transportation in China. After constructing ITN (see Fig. 4) based on high-speed trains and highways data, the number of links *k*_*i*_ is recorded for each node and the degree distribution *P*(*k*) is obtained (Fig. S1(a) in SI). The average degree 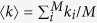 and the clustering coefficient 
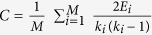
 are calculated, where *E*_*i*_ is the number of links connecting the *k*_*i*_ neighbors of node *i*^52^.

For the weights in Eq. (2), we record the actual populations in each node and reduce them to *N*_*i*_ in units of 5000 and the distances *r*_*ij*_ between pairs of nodes in *km* according to the China official website. The frequency of high-speed trains *S*_*ij*_ is obtained based on the routes and schedules of all high-speed trains. For each route that originates from a city *A* and terminates at a city *B*, we record the cities, say *A*, *C*1, *C*2, *C*3, *B*, served along the route and the number of services *m*_*s*_ per day. Then, all *S*_*ij*_, i.e. *S*_*A*,*C*1_, *S*_*C*1,*C*2_, *C*_*C*2,*C*3_, and *S*_*C*3, *B*_, are augmented by *m*_*s*_. Data for all routes give the final *S*_*ij*_ that go into Eq. (2) for the weights of the links *W*_*ij*_ and Eq. (3) for the weights of the nodes *W*_*i*_ (see Table S1 in SI).

### Journeys on ITN

For a journey that starts from the home city, the path of the shortest travel time to the destination is chosen. For a single type of links, i.e., *v*_*s*_ = *v*_*f*_, the path of shortest travel time coincides with the shortest path. In ITN with *v*_*s*_ < *v*_*f*_, the shortest paths are generally different from the paths of shortest time. As *v*_*f*_ > *v*_*s*_, selected paths will involve railways as much as possible. It is convenient to discretize the journeys. The distance *r*_*ij*_ between two neighboring nodes *i* and *j* are divided into *τ*_*ij*_ time steps. At each time step, 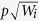 agents at node *i* become travellers. The destinations are chosen according to Eq. (5). The journeys are carried out as follows:

1. For every path between the home city *i* and destination *j*, the sum of *τ*_*ij*_ along the path is obtained. The path of shortest time is the one with the smallest sum.
2. Paths originated from different cities to different destinations may partially overlap. Therefore, in the intermediate nodes (cities) in a journey, some travellers may come in and other travellers may leave.
3. Upon arrival at the destination, an agent stays 5 time steps before the return journey begins.

Initially, the segments 1 ≤ *k*_*r*_ ≤ *τ*_*ij*_ on the links are empty and they will be occupied only when agents travel. For a node *i*, there are 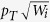 new travellers starting their journeys in the steady state, making a total 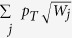 new travellers. Each of them has the chance 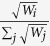 of choosing node *i* as the destination, giving a total 
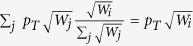
 agents arriving per time step in the steady state.

### Epidemic spreading measurement on ITN

In the SIS dynamics, we distinguish infections among non-travellers in the cities and among travellers in the same segment of a link with infectious rates *β*_1_ and *β*_2_, respectively. As travellers on trains/buses are densely packed, *β*_2_* *> *β*_1_^47^. An agent is a traveller and non-traveller at different times. When he is a non-traveller in a city, he is exposed to an infectious rate of *β*_1_. Once he is on a journey, he is exposed to an infectious rate of *β*_2_ during each segment of his journey, regardless of the segment being in the middle of a link or a passing-by city. Only travelling agents in the same segment *k*_*r*_ (1 ≤ *k*_*r*_ ≤ *τ*_*ij*_) towards the same direction can infect each other. Thus, SIS on ITN accounts for the continual exchanges of agents on trains and buses due to partial overlaps of agents’ journeys and the spread of a diseases through journeys. A susceptible non-traveller at node *i* will be infected by the rate 1−(1−*β*_1_)^*n*^_*i,I*_ when he is in contact with *n*_*i,I*_ infected agents. A susceptible traveller at a segment *k*_*r*_ of a link will be infected by the rate 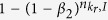 when he is in contact with 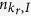 infected agents. Each infected agent recovers with a rate *μ*. The fraction *ρ*_*I*_ of infected agents is obtained by 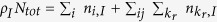, where 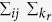 is over all the segments in all links in both travelling directions and *N*_*tot*_ is the total population.

### An approximate theoretical analysis

We make a qualitative analysis of the key behavior and illustrate that the dependence of *ρ*_*I*_ on the model parameters in ITN can be captured by mean-field considerations. Let there be *M* cities. There are 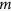 pairs of cities that the journey between which is all on high-speed trains. The mean number of sections 〈*τ*〉 in a link is *τ*_*s*_ = *int*(*s*/*v*_*s*_) + 1 for highway links and *τ*_*f*_ = *int*(*s*/*v*_*f*_) + 1 for railway links, where 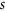 is the mean distance between neighbouring nodes. There are altogether


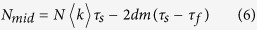


sections on the links, with *d* being the mean shortest path length between two nodes. It follows that *N*_*mid*_ decreases with *m*.

There are two processes in one time step: infection and motion. For the step *t *→ (*t* + 1), SIS processes take place in the time interval *t*^+^ → (*t* + 1)^−^ and the motion occurs at (*t* + 1). At a node 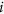, there are *n*_*i,s*_ susceptible and *n*_*i,I*_ infected agents and *n*_*i*_ = *n*_*i,S*_ + *n*_*i,I*_. Similarly, there are *n*_*α*,*S*_ susceptible and *n*_*α*,*I*_ infected agents at a section *α* of a link, with 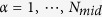 and 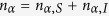. The dynamics of the infected agents can be described by


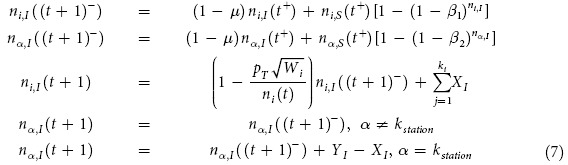


where *X*_*I*_ accounts for infected agents arriving at the destination or at home, *Y*_*I*_ represents infected agents starting a journey, *k*_*station*_ are nodes where agents switch means of transportation, and 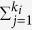 is over the *k*_*i*_ links to node *i*.

The time evolution of *ρ*_*I*_ is given by 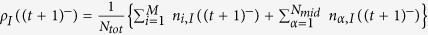 and thus


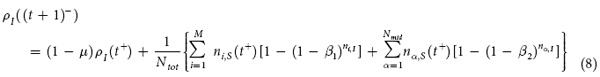


where *N*_*tot*_ is the total population. The set of equations can be iterated in time for the steady state. Further generalizations of ITN can be treated accordingly.

Based on Eq. (8), we make the following observations:

1. *For β*_1_ = *β*_2_: As *n*_*i*_ >> *n*_*α*_, we readily have *n*_*i,I*_ >> *n*_*α,I*_ and the second term in Eq. (8) dominates. Thus, *ρ*_*I*_ in Fig. 5(b) comes mostly from infections at the nodes.

2. *For β*_1_ ≠ *β*_2_ *and β*_1_ > *β*_1*c*_: Infections at the nodes give *ρ* ≠ 0, but the third term in Eq. (8) becomes important when *β*_2_ > *β*_1_ and *β*_2_ > *β*_2*c*_. This gives the behaviour in Fig. 6(a).

3. *For β*_1_ ≠ *β*_2_ *with β*_1_ < *β*_1*c*_: Infections at the nodes alone cannot sustain *ρ*_*I*_. Infections on journeys dominate and *ρ*_*I*_ becomes finite at *β*_2_ = *β*_2*c*_, independent of *β*_1_ (see Fig. 5d). It follows from the equation for *n*_*α*,*I*_((t + 1)^−^) that


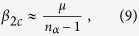


indicating that *β*_2*c*_ is inversely proportional to the mean number of agents travelling in a segment of a link *n*_*α*_.

4. *For different m*: The third term in Eq. (8) indicates that *ρ*_*I*_ ∝ *N*_*mid*_. As *N*_*mid*_ decreases with *m* (see Eq. 6), *ρ*_*I*_ also drops with increasing *m* and high-speed railways tend to prevent epidemics by shortening travel times. One should note that this captures one effect of having faster transportation. However, an opposite effect of inducing more travellers poses a risk.

## Additional Information

**How to cite this article**: Ruan, Z. *et al.* Integrated travel network model for studying epidemics: Interplay between journeys and epidemic. *Sci. Rep.* **5**, 11401; doi: 10.1038/srep11401 (2015).

## Supplementary Material

Supplementary Information
