# Supplementary material for: Integrated travel network model for studying epidemics: Interplay between journeys and epidemic
Source: Sci Rep. 2015 Jun 15;5:11401. doi: 10.1038/srep11401 (PMC4466778; doi:10.1038/srep11401)
Supplement: Supplementary Information [file srep11401-s1.pdf]

Supplementary Information: Integrated travel network model for studying epidemics: Interplay between journeys and epidemic

Zhongyuan Ruan, Chaoqing Wang, Pak Ming Hui, and Zonghua Liu

I. SUPPLEMENTARY FIGURES AND TABLES

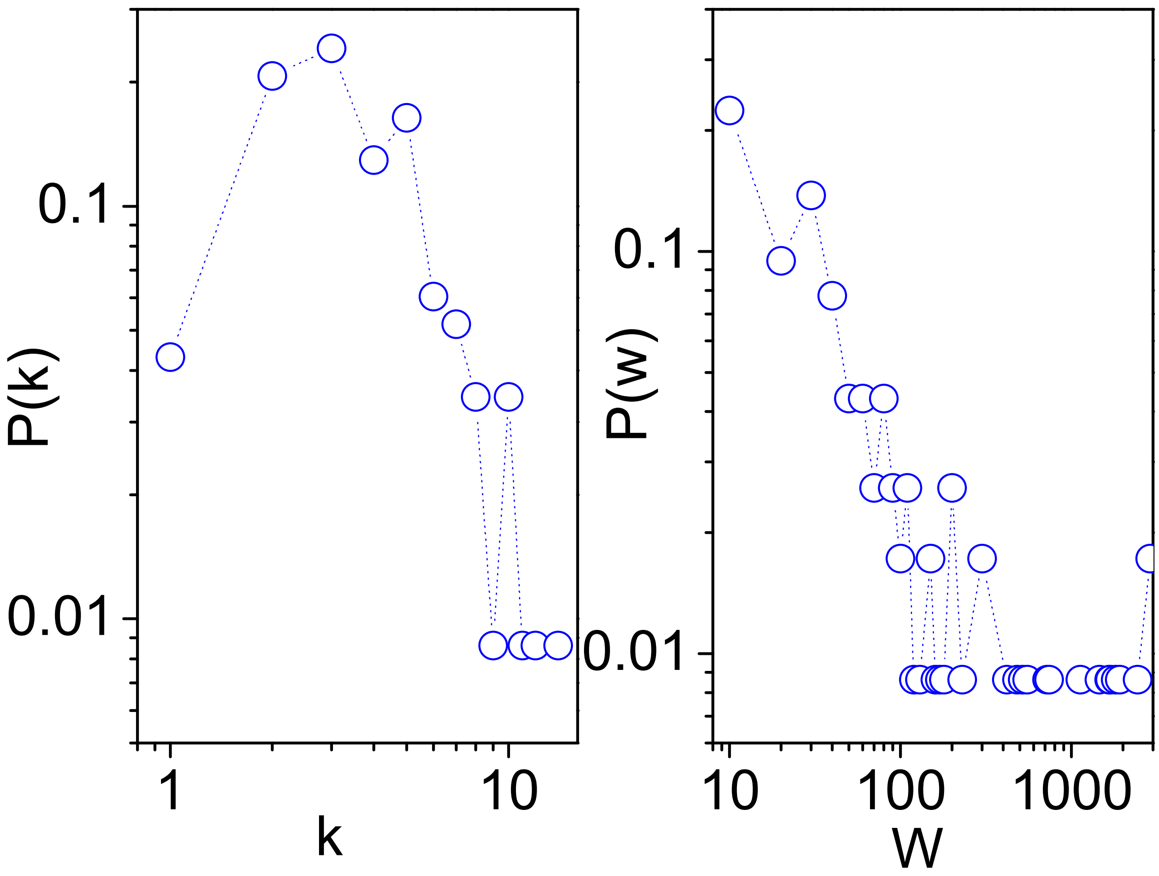

FIG. 1: (color online.) **Degree and weight distributions of Integrated Travel Network of 116 China highly populated cities**. (a) Degree distribution of ITN with an average degree $\langle k \rangle = 4.25$ and clustering coefficient $C = 0.35$, indicating the network is highly clustered. (b) Weight distribution of ITN.

To test the sensitivity to different ways of scaling the population, we use different units beside the one of 5000 in the simulation. Firstly, we calculate the dependence of the number of all non-travelers $\sum n_i$ on f by using different units, where f denotes the fraction of agents in the network that will start a journey in a time step. Fig. S2(a) shows the results, where the inset represents the normalized results by their first value, i.e. the value of $\sum n_i$ with $f = 0$. It is easy to see that the four curves are overlapped, indicating that the unit used to measure the population is not very important. We also noticed that they have a common linear part for $f \leq 0.01$. This result can be explained as follows. Before scaling, the number of agents at node i is N_i, the total number of agents is N_{tot}, the weight of a link is $W_{ij} \sim N_i N_j / r_{ij}^2$, node weight is $W_i \sim \sum_j W_{ij}$ and the number of agents starting to travel at each time step at node-i is $N_i^T = p_T \sqrt{W_i}$, where $p_T = f N_{tot} / \sum \sqrt{W_i}$. Now we rescale N_i by a factor k, the corresponding parameters becomes $N_i' = kN_i, W_{ij}' = k^2 W_{ij}$, $W_i' = k^2 W_i, p_T' = p_T$ and $N_i^{T'} = kN_i^T$. Thus, when the number of agents at node i increases (or decreases) by a factor k, the number of traveling agents will increase (decrease) by a factor k correspondingly. This makes the dynamic behavior similar before and after rescaling. Fig. S2(b) shows how the infected density varies with β_2 for different scaling units.

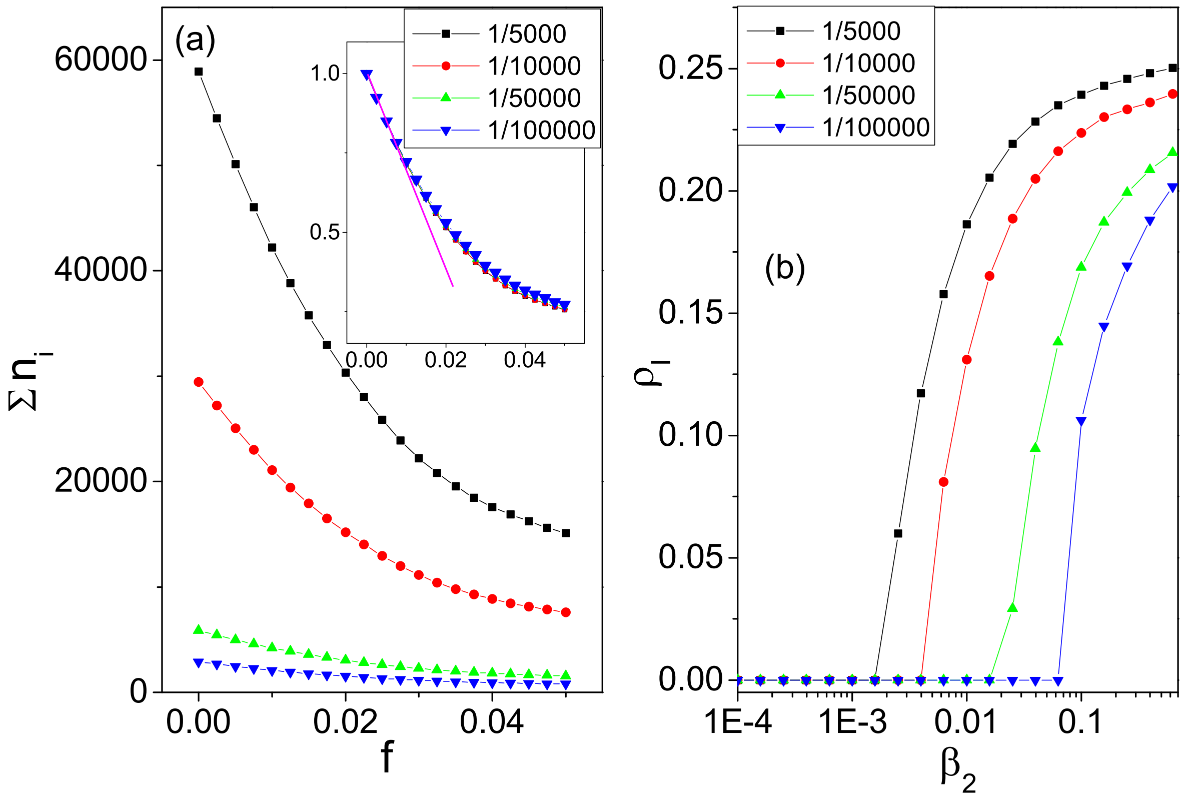

FIG. 2: (color online.) **Sensitivity to population scaling**. (a) Dependence of the number of all non-travellers $\sum_i n_i$ on f for different scaling units, where f is the fraction of agents in the network who will start a journey in time step. The inset represents the normalized results by their first value, i.e. the value of $\sum n_i$ with $f = 0$. The normalized four curves are overlapped, indicating that the unit used to measure the population is not very important. It is also noticed that they have a common linear part for $f \leq 0.01$, indicating it is a reasonable range. An increase in f results in more agents travelling along the links and thus a smaller $\sum_i n_i$ at nodes. When f is too large, too many agents are travelling and the unreasonable situations arise where the number of non-travellers at a node i becomes less than $p_T \sqrt{W_i}$, giving rise to a deviation from a linear dependence. A reasonable range of f is thus $f \leq 0.01$. (b) Infected density as a function of β_2 for different scaling units, where $\beta_1 = 2 \times 10^{-5}$ and $f = 0.01$.

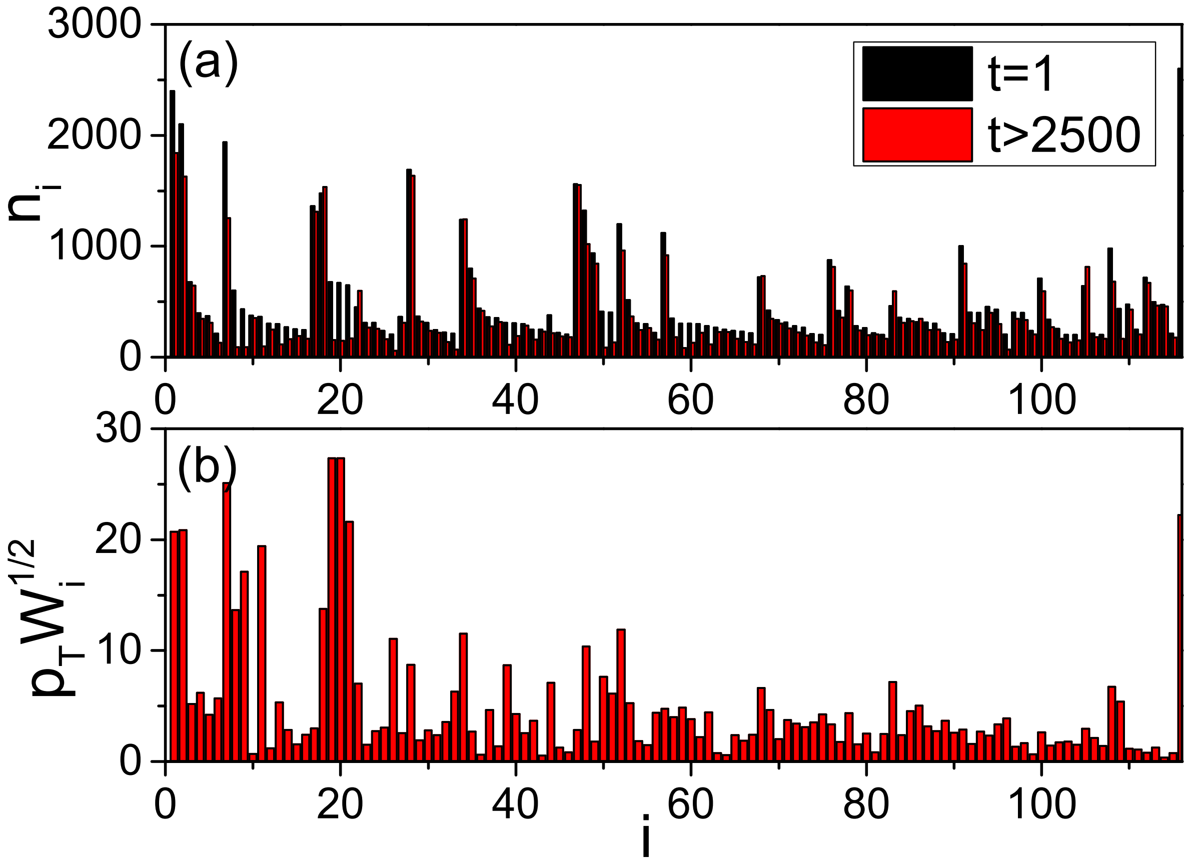

FIG. 3: (color online.) **Distribution of agents in steady state with** $f = 0.01$. (a) n_i at each of the nodes i for the 116 cities. The $t = 1$ case is the initial distribution of N_i and $t > 2500$ case represents the steady state. The difference between the initial and steady state values is not strictly proportional to the initial value among the cities, as factors of convenience in travelling has been considered. (b) The number of new travellers $p_T\sqrt{W_i}$ in a time step for the 116 cities. Comparing (b) with (a), (b) is approximately 1% of (a) which is consistent with $f = 0.01$.

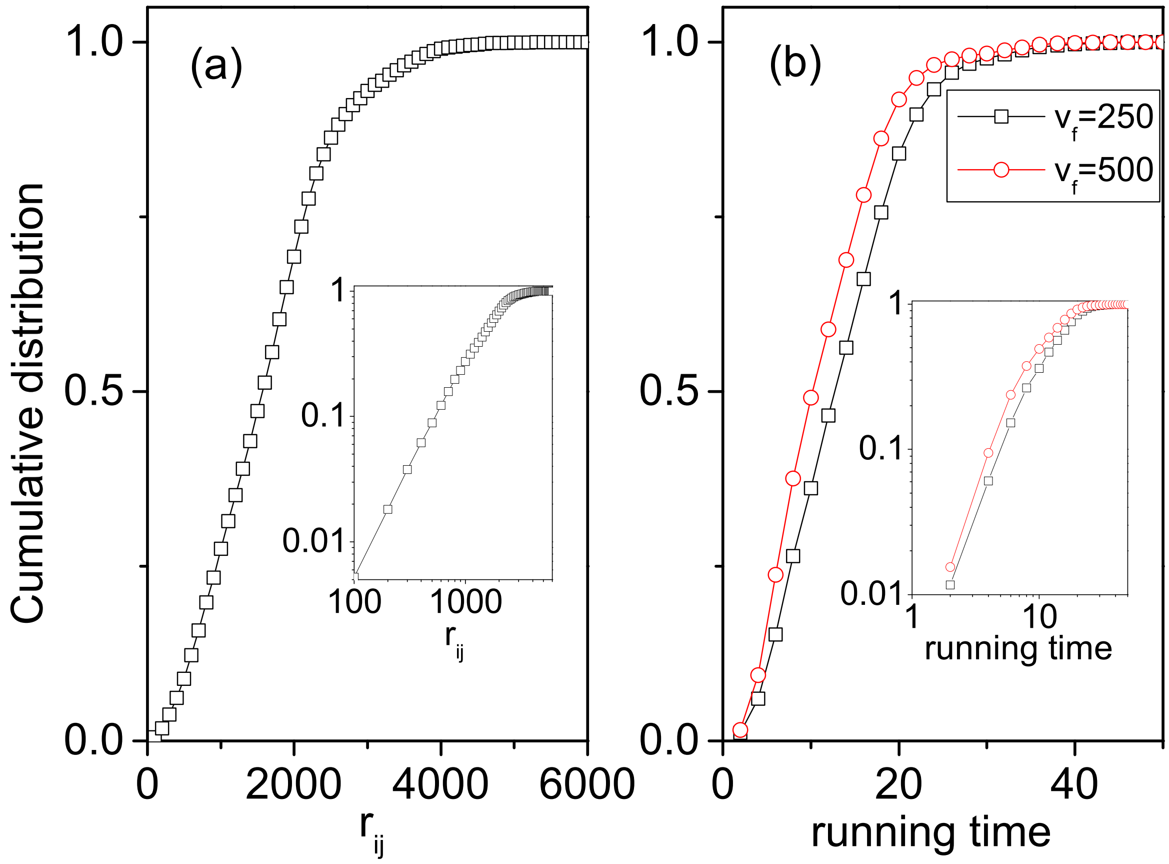

FIG. 4: (color online.) **Shortest paths and shortest travel times**. (a) Cumulative distribution of the shortest distances between any two nodes in ITN. The inset gives the plot in log-log scale. (b) Cumulative distribution of the shortest travel times between any two nodes in ITN. The inset gives the plot in log-log scale. Travel times take into account of the means of transportation. The two curves represent two values of train speed $v_f = 250$ and 500. Comparing the insets of (a) and (b), it is approximately a straight line in (a) but a curve in (b).

TABLE I: Populations N_i and weights W_i of the 116 highly populated cities in China with populations over one million.

node i	city	N_i/5000	W_i	node i	city	N_i/5000	W_i
1	Beijing	2400	1658.33	59	Suining	304	91.83
2	Tianjing	2100	1678.91	60	Zigong	302	55.85
3	Shijiazhuang	678	104.64	61	Luzhou	300	18.71
4	Tangshan	396	149.02	62	Neijiang	280	75.41
5	Handan	370	68.32	63	Bazhong	266	2.12
6	Baoding	212	124.99	64	Guang'an	250	1.30
7	Guangzhou	1936	2433.08	65	Mianyang	236	21.72
8	Shenzhen	600	719.14	66	Leshan	230	13.72
9	Dongguan	432	1129.67	67	Ziyang	216	22.68
10	Shantou	376	1.76	68	Zhengzhou	720	169.21
11	Foshan	364	1456.75	69	Luoyang	420	83.24
12	Zhanjiang	302	5.35	70	Nanyang	336	15.46
13	Zhongshan	300	109.67	71	Shangqiu	314	54.38
14	Jiangmen	270	31.14	72	Xinyang	280	45.26
15	Maoming	252	9.41	73	Luohe	268	37.07
16	Huizhou	244	22.50	74	Anyang	210	48.04
17	Chongqing	1360	34.60	75	Xinxiang	200	69.54
18	Nanjing	1478	733.01	76	Taiyuan	876	43.07
19	Wuxi	676	2887.38	77	Datong	416	12.07
20	Suzhou	670	2885.97	78	Changsha	636	73.53
21	Changzhou	650	1803.48	79	Changde	280	9.40
22	Xuzhou	450	191.37	80	Yiyang	262	24.90
23	Yancheng	308	8.99	81	Yongzhou	216	2.55
24	Suqian	308	28.90	82	Hengyang	200	24.10
25	Yangzhou	236	35.96	83	Hefei	460	197.67
26	Zhenjiang	204	471.84	84	Fuyang	356	22.11
27	Huai'an	362	25.41	85	Lu'an	344	79.29
28	Wuhan	1690	292.81	86	Suzhou	316	98.39
29	Xiangyang	366	14.25	87	Huainan	312	38.80
30	Suizhou	310	30.15	88	Haozhou	304	28.58
31	Yichang	246	21.58	89	Huaibei	216	52.09
32	Jingzhou	224	48.70	90	Wuhu	210	25.75
33	E'zhou	214	153.84	91	Ha'erbin	1000	31.98
34	Jinan	1240	513.76	92	Qiqiha'er	402	9.66
35	Qingdao	800	28.02	93	Daqing	400	27.87
36	Yantai	440	1.47	94	Fuzhou	452	21.38
37	Zaozhuang	360	82.83	95	Xiamen	430	43.45
38	Linyi	352	7.09	96	Quanzhou	204	58.79
39	Tai'an	310	291.15	97	Baotou	402	6.68
40	Weifang	306	71.19	98	Huhehaote	400	10.82
41	Heze	300	25.04	99	Chifeng	236	1.57
42	Laiwu	250	52.33	100	Nanning	710	26.38
43	Rizhao	250	1.18	101	Guigang	340	7.97
44	Zibo	378	193.81	102	Qinzhou	256	11.72
45	Jining	218	6.16	103	Laibin	202	12.43
46	Liaocheng	206	2.72	104	Liuzhou	200	8.77
47	Xi'an	1560	31.28	105	Nanchang	642	33.31
48	Shenyang	1320	415.72	106	Fuzhou	212	17.33
49	Dalian	936	12.62	107	Yichun	202	7.54
50	Anshan	410	225.89	108	Changchun	978	175.45

Continue

node i	city	N_i/5000	W_i	node i	city	N_i/5000	W_i
51	Fushun	404	144.09	109	Jilin	436	112.80
52	Hangzhou	1200	544.86	110	Lanzhou	476	5.04
53	Ningbo	516	106.41	111	Tianshui	248	4.57
54	Taizhou	306	13.09	112	Kunming	716	2.53
55	Wenzhou	300	8.44	113	Guiyang	496	6.03
56	Huzhou	218	74.09	114	Wulumuqi	470	0.08
57	Chengdu	1120	86.73	115	Xining	214	2.12
58	Nanchong	350	62.12	116	Shanghai	2600	1911.58

TABLE II: Distances r_{ij} in kilometers and weights S_{ij} of all the links, where the nodes marked with (R) are cities served by high-speed trains.

link $i \to j$	city-city	r_{ij}	S_{ij}	link $i \to j$	city-city	r_{ij}	S_{ij}
$1 \to j = 2$	Beijing(R)-Tianjing(R)	137	84	$55 \to j = 54$	Wenzhou(R)-Taizhou(R)	481	22
$1 \to j = 4$	Beijing(R)-Tangshan(R)	200	7	$55 \to j = 94$	Wenzhou(R)-Fuzhou(R)	294	23
$1 \to j = 6$	Beijing(R)-Baoding(R)	152	34	$56 \to j = 18$	Huzhou-Nanjing	205	0
$1 \to j = 34$	Beijing(R)-Jinan(R)	495	38	$56 \to j = 52$	Huzhou-Hangzhou	81	0
$1 \to j = 48$	Beijing(R)-Shenyang(R)	889	5	$56 \to j = 90$	Huzhou-Wuhu	211	0
$1 \to j = 77$	Beijing-Datong	374	0	$56 \to j = 116$	Huzhou-Shanghai	149	0
$1 \to j = 98$	Beijing-Huhehaote	659	0	$57 \to j = 59$	Chengdu(R)-Suining(R)	148	7
$2 \to j = 1$	Tianjing(R)-Beijing(R)	137	84	$57 \to j = 65$	Chengdu-Mianyang	115	0
$2 \to j = 4$	Tianjing(R)-Tangshan(R)	123	2	$57 \to j = 66$	Chengdu-Leshan	137	0
$2 \to j = 6$	Tianjing-Baoding	273	0	$57 \to j = 67$	Chengdu-Ziyang	122	0
$2 \to j = 34$	Tianjing(R)-Jinan(R)	357	30	$57 \to j = 112$	Chengdu-Kunming	1100	0
$2 \to j = 44$	Tianjing-Zibo	467	0	$58 \to j = 17$	Nanchong-Chongqing	234	0
$3 \to j = 5$	Shijiazhuang(R)-Handan(R)	165	16	$58 \to j = 59$	Nanchong(R)-Suining(R)	67	5
$3 \to j = 6$	Shijiazhuang(R)-Baoding(R)	131	34	$58 \to j = 63$	Nanchong-Bazhong	219	0
$3 \to j = 34$	Shijiazhuang-Jinan	298	0	$58 \to j = 64$	Nanchong-Guang'an	270	0
$3 \to j = 74$	Shijiazhuang(R)-Anyang(R)	225	6	$58 \to j = 110$	Nanchong-Lanzhou	1370	0
$3 \to j = 75$	Shijiazhuang(R)-Xinxiang(R)	332	2	$59 \to j = 17$	Suining(R)-Chongqing(R)	463	4
$3 \to j = 76$	Shijiazhuang(R)-Taiyuan(R)	231	18	$59 \to j = 57$	Suining(R)-Chengdu(R)	148	7
$4 \to j = 1$	Tangshan(R)-Beijing(R)	200	7	$59 \to j = 58$	Suining(R)-Nanchong(R)	67	5
$4 \to j = 2$	Tangshan(R)-Tianjing(R)	123	2	$59 \to j = 62$	Suining-Neijiang	367	0
$4 \to j = 48$	Tangshan(R)-Shenyang(R)	566	10	$59 \to j = 65$	Suining-Mianyang	263	0
$5 \to j = 3$	Handan(R)-Shijiazhuang(R)	165	16	$60 \to j = 62$	Zigong-Neijiang	39	0
$5 \to j = 46$	Handan-Tangshan	435	0	$60 \to j = 112$	Zigong-Kunming	913	0
$5 \to j = 68$	Handan(R)-Zhengzhou(R)	247	5	$61 \to j = 17$	Luzhou-Chongqing	203	0
$5 \to j = 74$	Handan(R)-Anyang(R)	60	3	$61 \to j = 62$	Luzhou-Neijiang	102	0
$5 \to j = 75$	Handan(R)-Xinxiang(R)	167	4	$61 \to j = 113$	Luzhou-Guiyang	447	0
$6 \to j = 1$	Baoding(R)-Beijing(R)	152	34	$62 \to j = 17$	Neijiang-Chongqing	285	0
$6 \to j = 2$	Baoding-Tianjing	273	0	$62 \to j = 59$	Neijiang-Suining	367	0
$6 \to j = 3$	Baoding(R)-Shijiazhuang(R)	131	34	$62 \to j = 60$	Neijiang-Zigong	39	0
$7 \to j = 8$	Guangzhou(R)-Shenzhen(R)	147	41	$62 \to j = 61$	Neijiang-Luzhou	102	0
$7 \to j = 9$	Guangzhou(R)-Dongguan(R)	90	107	$62 \to j = 67$	Neijiang-Ziyang	97	0
$7 \to j = 11$	Guangzhou-Foshan	22	0	$63 \to j = 58$	Bazhong-Nanchong	219	0
$7 \to j = 13$	Guangzhou-Zhongshan	86	0	$63 \to j = 110$	Bazhong-Lanzhou	822	0
$7 \to j = 15$	Guangzhou-Maoming	371	0	$64 \to j = 31$	Guang'an-Yichang	770	0
$7 \to j = 16$	Guangzhou-Huizhou	148	0	$64 \to j = 58$	Guang'an-Nanchong	270	0
$7 \to j = 78$	Guangzhou(R)-Changsha(R)	707	34	$65 \to j = 47$	Mianyang-Xi'an	727	0
$7 \to j = 82$	Guangzhou(R)-Hengyang(R)	521	43	$65 \to j = 57$	Mianyang-Chengdu	115	0
$7 \to j = 100$	Guangzhou-Nanning	809	0	$65 \to j = 59$	Mianyang-Suining	263	0
$7 \to j = 102$	Guangzhou-Qinzhou	938	0	$66 \to j = 57$	Leshan-Chengdu	137	0
$7 \to j = 105$	Guangzhou-Nanchang	900	0	$67 \to j = 57$	Ziyang-Chengdu	122	0
$8 \to j = 7$	Shenzhen(R)-Guangzhou(R)	147	41	$67 \to j = 62$	Ziyang-Neijiang	97	0
$8 \to j = 9$	Shenzhen(R)-Dongguan(R)	57	107	$68 \to j = 5$	Zhengzhou(R)-Handan(R)	247	5
$8 \to j = 10$	Shenzhen-Shantou	502	0	$68 \to j = 18$	Zhengzhou(R)-Nanjing(R)	697	1
$9 \to j = 7$	Dongguan(R)-Guangzhou(R)	90	107	$68 \to j = 28$	Zhengzhou(R)-Wuhan(R)	514	12
$9 \to j = 8$	Dongguan(R)-Shenzhen(R)	57	107	$68 \to j = 41$	Zhengzhou-Heze	298	0
$10 \to j = 8$	Shantou-Shenzhen	502	0	$68 \to j = 69$	Zhengzhou(R)-Luoyang(R)	124	21
$10 \to j = 16$	Shantou-Huizhou	387	0	$68 \to j = 70$	Zhengzhou-Nanyang	369	0
$10 \to j = 95$	Shantou-Xiamen	787	0	$68 \to j = 71$	Zhengzhou(R)-Shangqiu(R)	203	4
$11 \to j = 7$	Foshan-Guangzhou	22	0	$68 \to j = 72$	Zhengzhou(R)-Xinyang(R)	302	9
$11 \to j = 15$	Foshan-Maoming	349	0	$68 \to j = 73$	Zhengzhou(R)-Luohe(R)	140	13
$12 \to j = 15$	Zhanjiang-Maoming	122	0	$68 \to j = 74$	Zhengzhou(R)-Anyang(R)	187	7
$12 \to j = 102$	Zhanjiang-Qinzhou	567	0	$68 \to j = 75$	Zhengzhou(R)-Xinxiang(R)	80	11

Continue

$13 \to j = 7$	Zhongshan-Guangzhou	86	0	$68 \to j = 76$	Zhengzhou-Taiyuan	577	0
$13 \to j = 14$	Zhongshan-Jiangmen	51	0	$69 \to j = 47$	Luoyang(R)-Xi'an(R)	387	20
$14 \to j = 13$	Jiangmen-Zhongshan	51	0	$69 \to j = 68$	Luoyang(R)-Zhengzhou(R)	124	21
$15 \to j = 7$	Maoming-Guangzhou	371	0	$69 \to j = 70$	Luoyang(R)-Nanyang(R)	256	0
$15 \to j = 11$	Maoming-Foshan	349	0	$69 \to j = 73$	Luoyang-Luohe	264	0
$15 \to j = 12$	Maoming-Zhanjiang	122	0	$69 \to j = 76$	Luoyang-Taiyuan	792	0
$16 \to j = 7$	Huizhou-Guangzhou	148	0	$70 \to j = 29$	Nanyang-Xiangyang	138	0
$16 \to j = 10$	Huizhou-Shantou	387	0	$70 \to j = 41$	Nanyang-Heze	668	0
$16 \to j = 105$	Huizhou-Nanchang	808	0	$70 \to j = 47$	Nanyang-Xi'an	452	0
$16 \to j = 106$	Huizhou-Fuzhou	780	0	$70 \to j = 68$	Nanyang-Zhengzhou	369	0
$17 \to j = 31$	Chongqing-Yichang	776	0	$70 \to j = 69$	Nanyang-Luoyang	256	0
$17 \to j = 47$	Chongqing-Xi'an	790	0	$70 \to j = 72$	Nanyang-Xinyang	203	0
$17 \to j = 58$	Chongqing-Nanchong	234	0	$71 \to j = 22$	Shangqiu(R)-Xuzhou(R)	146	4
$17 \to j = 59$	Chongqing(R)-Suining(R)	463	4	$71 \to j = 41$	Shangqiu-Heze	95	0
$17 \to j = 61$	Chongqing-Luzhou	203	0	$71 \to j = 68$	Shangqiu(R)-Zhengzhou(R)	203	4
$17 \to j = 62$	Chongqing-Neijiang	285	0	$71 \to j = 73$	Shangqiu-Luohe	343	0
$17 \to j = 79$	Chongqing-Changde	1028	0	$71 \to j = 88$	Shangqiu-Haozhou	74	0
$17 \to j = 113$	Chongqing-Guiyang	463	0	$71 \to j = 89$	Shangqiu-Huaibei	208	0
$18 \to j = 19$	Nanjing(R)-Wuxi(R)	175	13	$72 \to j = 28$	Xinyang(R)-Wuhan(R)	212	18
$18 \to j = 20$	Nanjing(R)-Suzhou(R)	217	7	$72 \to j = 30$	Xinyang-Suizhou	244	0
$18 \to j = 21$	Nanjing(R)-Changzhou(R)	136	34	$72 \to j = 68$	Xinyang(R)-Zhengzhou(R)	302	9
$18 \to j = 22$	Nanjing(R)-Xuzhou(R)	348	28	$72 \to j = 70$	Xinyang-Nanyang	203	0
$18 \to j = 24$	Nanjing-Suqian	249	0	$72 \to j = 73$	Xinyang(R)-Luohe(R)	162	9
$18 \to j = 25$	Nanjing-Yangzhou	101	0	$72 \to j = 85$	Xinyang-Lu'an	252	0
$18 \to j = 26$	Nanjing(R)-Zhenjiang(R)	64	60	$73 \to j = 28$	Luohe(R)-Wuhan(R)	374	4
$18 \to j = 27$	Nanjing-Huai'an	445	0	$73 \to j = 68$	Luohe(R)-Zhengzhou(R)	140	13
$18 \to j = 56$	Nanjing-Huzhou	205	0	$73 \to j = 69$	Luohe-Luoyang	264	0
$18 \to j = 68$	Nanjing(R)-Zhengzhou(R)	697	1	$73 \to j = 71$	Luohe-Shangqiu	343	0
$18 \to j = 83$	Nanjing(R)-Hefei(R)	156	26	$73 \to j = 72$	Luohe-Xinyang	162	9
$18 \to j = 86$	Nanjing(R)-Suzhou(R)	274	11	$74 \to j = 3$	Anyang(R)-Shijiazhuang(R)	225	6
$18 \to j = 90$	Nanjing-Wuhu	125	0	$74 \to j = 5$	Anyang(R)-Handan(R)	60	3
$18 \to j = 116$	Nanjing(R)-Shanghai(R)	301	14	$74 \to j = 68$	Anyang(R)-Zhengzhou(R)	187	7
$19 \to j = 18$	Wuxi(R)-Nanjing(R)	175	13	$74 \to j = 75$	Anyang(R)-Xinxiang(R)	107	5
$19 \to j = 20$	Wuxi(R)-Suzhou(R)	42	79	$75 \to j = 3$	Xinxiang(R)-Shijiazhuang(R)	332	2
$19 \to j = 21$	Wuxi(R)-Changzhou(R)	39	69	$75 \to j = 5$	Xinxiang(R)-Handan(R)	167	4
$19 \to j = 23$	Wuxi-Yancheng	497	0	$75 \to j = 41$	Xinxiang-Heze	175	0
$19 \to j = 26$	Wuxi(R)-Zhenjiang(R)	111	10	$75 \to j = 68$	Xinxiang(R)-Zhengzhou(R)	80	11
$19 \to j = 86$	Wuxi-Suzhou	449	0	$75 \to j = 74$	Xinxiang(R)-Anyang(R)	107	5
$19 \to j = 116$	Wuxi(R)-Shanghai(R)	126	19	$76 \to j = 3$	Taiyuan(R)-Shijiazhuang(R)	231	18
$20 \to j = 18$	Suzhou(R)-Nanjing(R)	217	7	$76 \to j = 47$	Taiyuan-Xi'an	679	0
$20 \to j = 19$	Suzhou(R)-Wuxi(R)	42	79	$76 \to j = 68$	Taiyuan-Zhengzhou	577	0
$20 \to j = 21$	Suzhou(R)-Changzhou(R)	81	10	$76 \to j = 69$	Taiyuan-Luoyang	792	0
$20 \to j = 23$	Suzhou-yancheng	539	0	$76 \to j = 77$	Taiyuan-Datong	355	0
$20 \to j = 116$	Suzhou(R)-Shanghai(R)	84	101	$77 \to j = 1$	Datong-Beijing	374	0
$21 \to j = 18$	Changzhou(R)-Nanjing(R)	136	34	$77 \to j = 76$	Datong-Taiyuan	355	0
$21 \to j = 19$	Changzhou(R)-Wuxi(R)	39	69	$77 \to j = 98$	Datong-Huhehaote	285	0
$21 \to j = 20$	Changzhou(R)-Suzhou(R)	81	10	$78 \to j = 7$	Changsha(R)-Guangzhou(R)	707	34
$21 \to j = 26$	Changzhou(R)-Zhenjiang(R)	72	45	$78 \to j = 28$	Changsha(R)-Wuhan(R)	413	61
$21 \to j = 116$	Changzhou(R)-Shanghai(R)	165	3	$78 \to j = 80$	Changsha-Yiyang	97	0

Continue

$22 \rightarrow j = 18$	Xuzhou(R)-Nanjing(R)	348	28	$78 \rightarrow j = 82$	Changsha(R)-Hengyang(R)	186	43
$22 \rightarrow j = 24$	Xuzhou-Suqian	121	0	$78 \rightarrow j = 105$	Changsha(R)-Nanchang (R)	419	2
$22 \rightarrow j = 34$	Xuzhou(R)-Jinan(R)	319	17	$78 \rightarrow j = 107$	Changsha(R)-Yichun(R)	199	1
$22 \rightarrow j = 37$	Xuzhou(R)-Zaozhuang(R)	80	15	$78 \rightarrow j = 113$	Changsha-Guiyang	949	0
$22 \rightarrow j = 39$	Xuzhou(R)-Tai'an(R)	351	3	$79 \rightarrow j = 17$	Changde-Chongqing	1028	0
$22 \rightarrow j = 71$	Xuzhou(R)-Shangqiu(R)	146	4	$79 \rightarrow j = 32$	Changde-Jingzhou	184	0
$22 \rightarrow j = 86$	Xuzhou(R)-Suzhou(R)	74	11	$79 \rightarrow j = 80$	Changde-Yiyang	101	0
$22 \rightarrow j = 87$	Xuzhou(R)-Huainan(R)	349	1	$80 \rightarrow j = 78$	Yiyang-Changsha	97	0
$22 \rightarrow j = 89$	Xuzhou-Huaibei	62	0	$80 \rightarrow j = 79$	Yiyang-Changde	101	0
$23 \rightarrow j = 19$	Yancheng-Wuxi	497	0	$81 \rightarrow j = 82$	Yongzhou-Hengyan	141	0
$23 \rightarrow j = 20$	Yancheng-Suzhou	539	0	$81 \rightarrow j = 104$	Yongzhou-Liuzhou	397	0
$23 \rightarrow j = 27$	Yancheng-Huaian	123	0	$81 \rightarrow j = 113$	Yongzhou-Guiyang	991	0
$23 \rightarrow j = 43$	Yancheng-Rizhao	1039	0	$82 \rightarrow j = 7$	Hengyang(R)-Guangzhou(R)	521	43
$24 \rightarrow j = 18$	Suqian-Nanjing	249	0	$82 \rightarrow j = 78$	Hengyang(R)-Changsha(R)	186	43
$24 \rightarrow j = 22$	Suqian-Xuzhou	121	0	$82 \rightarrow j = 81$	Hengyang-Yongzhou	141	0
$24 \rightarrow j = 27$	Suqian-Huai'an	96	0	$82 \rightarrow j = 113$	Hengyang-Guiyang	1031	0
$25 \rightarrow j = 18$	Yangzhou-Nanjing	101	0	$83 \rightarrow j = 18$	Hefei(R)-Nanjing(R)	156	26
$25 \rightarrow j = 26$	Yangzhou-Zhenjiang	165	0	$83 \rightarrow j = 33$	Hefei-E'zhou	460	0
$26 \rightarrow j = 18$	Zhenjiang(R)-Nanjing(R)	64	60	$83 \rightarrow j = 84$	Hefei-Fuyang	221	0
$26 \rightarrow j = 19$	Zhenjiang(R)-Wuxi(R)	111	10	$83 \rightarrow j = 85$	Hefei(R)-Lu'an(R)	92	14
$26 \rightarrow j = 21$	Zhenjiang(R)-Changzhou(R)	72	45	$83 \rightarrow j = 86$	Hefei-Suzhou	221	0
$26 \rightarrow j = 25$	Zhenjiang-Yangzhou	165	0	$83 \rightarrow j = 87$	Hefei(R)-Huainan(R)	95	4
$27 \rightarrow j = 18$	Huai'an-Nanjing	445	0	$83 \rightarrow j = 90$	Hefei-Wuhu	141	0
$27 \rightarrow j = 23$	Huai'an-yancheng	123	0	$83 \rightarrow j = 105$	Hefei-Nanchang	462	0
$27 \rightarrow j = 24$	Huai'an-Suqian	96	0	$84 \rightarrow j = 83$	Fuyang-Hefei	221	0
$27 \rightarrow j = 38$	Huai'an-Linyi	204	0	$84 \rightarrow j = 85$	Fuyang-Lu'an	264	0
$27 \rightarrow j = 43$	Huai'an-Rizhao	699	0	$84 \rightarrow j = 87$	Fuyang-Huainan	126	0
$28 \rightarrow j = 30$	Wuhan(R)-Suizhou(R)	197	3	$84 \rightarrow j = 88$	Fuyang-Haozhou	104	0
$28 \rightarrow j = 31$	Wuhan-Yichang	394	0	$85 \rightarrow j = 28$	Luan(R)-Wuhan(R)	270	13
$28 \rightarrow j = 32$	Wuhan(R)-Jingzhou(R)	227	32	$85 \rightarrow j = 72$	Lu'an-Xinyang	252	0
$28 \rightarrow j = 33$	Wuhan(R)-Ezhou(R)	60	1	$85 \rightarrow j = 83$	Lu'an(R)-Hefei(R)	92	14
$28 \rightarrow j = 68$	Wuhan(R)-Zhengzhou(R)	514	12	$85 \rightarrow j = 84$	Lu'an-Fuyang	264	0
$28 \rightarrow j = 72$	Wuhan(R)-Xinyang(R)	212	18	$86 \rightarrow j = 18$	Suzhou(R)-Nanjing(R)	274	11
$28 \rightarrow j = 73$	Wuhan(R)-Luohe(R)	374	4	$86 \rightarrow j = 19$	Suzhou-Wuxi	449	0
$28 \rightarrow j = 78$	Wuhan(R)-Changsha(R)	413	61	$86 \rightarrow j = 22$	Suzhou(R)-Xuzhou(R)	74	11
$28 \rightarrow j = 85$	Wuhan(R)-Lu'an(R)	270	13	$86 \rightarrow j = 83$	Suzhou-Hefei	221	0
$28 \rightarrow j = 105$	Wuhan(R)-Nanchang(R)	337	5	$86 \rightarrow j = 87$	Suzhou-Huainan	176	0
$29 \rightarrow j = 30$	Xiangyang-Suizhou	180	0	$86 \rightarrow j = 88$	Suzhou-Haozhou	289	0
$29 \rightarrow j = 31$	Xiangyang-Yichang	251	0	$86 \rightarrow j = 89$	Suzhou-Huaibei	52	0
$29 \rightarrow j = 32$	Xiangyang-Jingzhou	207	0	$86 \rightarrow j = 116$	Suzhou-Shanghai	575	0
$29 \rightarrow j = 47$	Xiangyang-Xi'an	772	0	$87 \rightarrow j = 22$	Huainan(R)-Xuzhou(R)	349	1
$29 \rightarrow j = 70$	Xiangyang-Nanyang	138	0	$87 \rightarrow j = 83$	Huainan(R)-Hefei(R)	95	4
$30 \rightarrow j = 28$	Suizhou(R)-Wuhan(R)	197	3	$87 \rightarrow j = 84$	Huainan-Fuyang	126	0
$30 \rightarrow j = 29$	Suizhou-Xiangyang	180	0	$87 \rightarrow j = 86$	Huainan-Suzhou	176	0
$30 \rightarrow j = 72$	Suizhou-Xinyang	244	0	$88 \rightarrow j = 71$	Haozhou-Shangqiu	74	0
$31 \rightarrow j = 17$	Yichang-Chongqing	776	0	$88 \rightarrow j = 84$	Haozhou-Fuyang	104	0
$31 \rightarrow j = 28$	Yichang-Wuhan	394	0	$88 \rightarrow j = 86$	Haozhou-Suzhou	289	0
$31 \rightarrow j = 29$	Yichang-Xiangyang	251	0	$89 \rightarrow j = 22$	Huaibei-Xuzhou	62	0
$31 \rightarrow j = 32$	Yichang(R)-Jingzhou(R)	112	32	$89 \rightarrow j = 71$	Huaibei-Shangqiu	108	0
$31 \rightarrow j = 64$	Yichang-Guang'an	770	0	$89 \rightarrow j = 86$	Huaibei-Suzhou	52	0
$32 \rightarrow j = 28$	Jingzhou(R)-Wuhan(R)	227	32	$90 \rightarrow j = 18$	Wuhu-Nanjing	125	0
$32 \rightarrow j = 29$	Jingzhou-Xiangyang	207	0	$90 \rightarrow j = 56$	Wuhu-Huzhou	211	0
$32 \rightarrow j = 31$	Jingzhou(R)-Yichang(R)	112	32	$90 \rightarrow j = 83$	Wuhu-Hefei	141	0
$32 \rightarrow j = 79$	Jingzhou-Changde	184	0	$91 \rightarrow j = 93$	Ha'erbin-Daqing	159	0

Continue

$33 \to j = 28$	E'zhou(R)-Wuhan(R)	60	1	$91 \to j = 108$	Ha'erbin-Changchun	246	0
$33 \to j = 83$	E'zhou-Hefei	460	0	$92 \to j = 93$	Qiqiha'er-Daqing	129	0
$33 \to j = 105$	E'zhou(R)-Nanchang(R)	277	1	$93 \to j = 91$	Daqing-Ha'erbin	159	0
$34 \to j = 1$	Jinan(R)-Beijing(R)	495	38	$93 \to j = 92$	Daqing-Qiqiha'er	129	0
$34 \to j = 2$	Jinan(R)-Tianjing(R)	357	30	$93 \to j = 108$	Daqing-Changchun	405	0
$34 \to j = 3$	Jinan-Shijiazhuang	298	0	$94 \to j = 55$	Fuzhou(R)-Wenzhou(R)	294	23
$34 \to j = 22$	Jinan(R)-Xuzhou(R)	319	17	$94 \to j = 96$	Fuzhou(R)-Quanzhou(R)	174	68
$34 \to j = 37$	Jinan(R)-Zaozhuang(R)	224	9	$94 \to j = 106$	Fuzhou-Fuzhou	461	0
$34 \to j = 39$	Jinan(R)-Tai'an(R)	62	10	$95 \to j = 10$	Xiamen-Shantou	787	0
$34 \to j = 41$	Jinan-Heze	295	0	$95 \to j = 96$	Xiamen(R)-Quanzhou(R)	102	68
$34 \to j = 42$	Jinan-Laiwu	124	0	$96 \to j = 94$	Quanzhou(R)-Fuzhou(R)	174	68
$34 \to j = 44$	Jinan(R)-Zibo(R)	110	29	$96 \to j = 95$	Quanzhou(R)-Xiamen(R)	102	68
$34 \to j = 46$	Jinan-Liaocheng	332	0	$97 \to j = 47$	Baotou-Xi'an	899	0
$35 \to j = 40$	Qingdao(R)-Weifang(R)	183	31	$97 \to j = 98$	Baotou-Huhehaote	165	0
$35 \to j = 43$	Qingdao-Rizhao	738	0	$98 \to j = 1$	Huhehaote-Beijing	659	0
$36 \to j = 40$	Yantai-Weifang	314	0	$98 \to j = 47$	Huhehaote-Xi'an	1064	0
$36 \to j = 43$	Yantai-Rizhao	996	0	$98 \to j = 77$	Huhehaote-Datong	285	0
$37 \to j = 22$	Zaozhuang(R)-Xuzhou(R)	80	15	$98 \to j = 97$	Huhehaote-Baotou	165	0
$37 \to j = 34$	Zaozhuang(R)-Jinan(R)	224	9	$98 \to j = 99$	Huhehaote-Chifeng	932	0
$37 \to j = 39$	Zaozhuang(R)-Taian(R)	162	4	$99 \to j = 48$	Chifeng-Shenyang	574	0
$38 \to j = 27$	Linyi-Huai'an	204	0	$99 \to j = 98$	Chifeng-Huhehaote	932	0
$38 \to j = 39$	Linyi-Tai'an	400	0	$99 \to j = 108$	Chifeng-Changchun	672	0
$38 \to j = 42$	Linyi-Laiwu	162	0	$100 \to j = 7$	Nanning-Guangzhou	809	0
$39 \to j = 22$	Tai'an(R)-Xuzhou(R)	351	3	$100 \to j = 101$	Nanning-Guigang	174	0
$39 \to j = 34$	Tai'an(R)-Jinan(R)	62	10	$100 \to j = 102$	Nanning-Qinzhou	129	0
$39 \to j = 37$	Tai'an(R)-Zaozhuang(R)	162	4	$100 \to j = 103$	Nanning-Laibin	185	0
$39 \to j = 38$	Tai'an-Linyi	400	0	$100 \to j = 112$	Nanning-Kunming	828	0
$39 \to j = 42$	Tai'an-Laiwu	57	0	$100 \to j = 113$	Nanning-Guiyang	875	0
$40 \to j = 35$	Weifang(R)-Qingdao(R)	183	31	$101 \to j = 100$	Guigang-Nanning	174	0
$40 \to j = 36$	Weifang-Yantai	314	0	$102 \to j = 7$	Qinzhou-Guangzhou	938	0
$40 \to j = 44$	Weifang(R)-Zibo(R)	100	28	$102 \to j = 12$	Qinzhou-Zhanjiang	567	0
$41 \to j = 34$	Heze-Jinan	295	0	$102 \to j = 100$	Qinzhou-Nanning	129	0
$41 \to j = 45$	Heze-Jining	107	0	$103 \to j = 100$	Laibin-Nanning	185	0
$41 \to j = 68$	Heze-Zhengzhou	298	0	$103 \to j = 104$	Laibin-Liuzhou	70	0
$41 \to j = 70$	Heze-Nanyang	668	0	$104 \to j = 81$	Liuzhou-Yongzhou	397	0
$41 \to j = 71$	Heze-Shangqiu	95	0	$104 \to j = 103$	Liuzhou-Laibin	70	0
$41 \to j = 75$	Heze-Xinxiang	175	0	$104 \to j = 113$	Liuzhou-Guiyang	620	0
$42 \to j = 34$	Laiwu-Jinan	124	0	$105 \to j = 7$	Nanchang-Guangzhou	900	0
$42 \to j = 38$	Laiwu-Linyi	162	0	$105 \to j = 16$	Nanchang-Huizhou	808	0
$42 \to j = 39$	Laiwu-Tai'an	57	0	$105 \to j = 28$	Nanchang(R)-Wuhan(R)	337	5
$42 \to j = 44$	Laiwu-Zibo	138	0	$105 \to j = 33$	Nanchang(R)-E'zhou(R)	277	1
$43 \to j = 23$	Rizhao-yancheng	1039	0	$105 \to j = 52$	Nanchang(R)-Hangzhou(R)	644	3
$43 \to j = 27$	Rizhao-Huai'an	699	0	$105 \to j = 53$	Nanchang-Ningbo	778	0
$43 \to j = 35$	Rizhao-Qingdao	738	0	$105 \to j = 78$	Nanchang(R)-Changsha(R)	419	2
$43 \to j = 36$	Rizhao-Yantai	996	0	$105 \to j = 83$	Nanchang-Hefei	462	0
$43 \to j = 45$	Rizhao-Jining	348	0	$105 \to j = 106$	Nanchang-Fuzhou	90	0
$44 \to j = 2$	Zibo-Tianjing	467	0	$105 \to j = 107$	Nanchang-Yichun	220	0
$44 \to j = 34$	Zibo(R)-Jinan(R)	110	29	$106 \to j = 16$	Fuzhou-Huizhou	780	0
$44 \to j = 40$	Zibo(R)-Weifang(R)	100	28	$106 \to j = 94$	Fuzhou-Fuzhou	461	0
$44 \to j = 42$	Zibo-Laiwu	138	0	$106 \to j = 105$	Fuzhou-Nanchang	90	0
$45 \to j = 41$	Jining-Heze	107	0	$107 \to j = 52$	Yichun(R)-Hangzhou(R)	790	1
$45 \to j = 43$	Jining-Rizhao	348	0	$107 \to j = 78$	Yichun(R)-Changsha(R)	199	1
$46 \to j = 5$	Liaocheng-Handan	435	0	$107 \to j = 105$	Yichun-Nanchang	220	0
$46 \to j = 34$	Liaocheng-Jinan	332	0	$108 \to j = 48$	Changchun(R)-Shenyang(R)	303	22

Continue

$47 \to j = 17$	Xi'an-Chongqing	790	0	$108 \to j = 91$	Changchun-Ha'erbin	246	0
$47 \to j = 29$	Xi'an-Xiangyang	772	0	$108 \to j = 93$	Changchun-Daqing	405	0
$47 \to j = 65$	Xi'an-Mianyang	727	0	$108 \to j = 99$	Changchun-Chifeng	672	0
$47 \to j = 69$	Xi'an(R)-Luoyang(R)	387	20	$108 \to j = 109$	Changchun(R)-Jilin(R)	128	41
$47 \to j = 70$	Xi'an-Nanyang	452	0	$109 \to j = 48$	Jilin-Shenyang	464	0
$47 \to j = 76$	Xi'an-Taiyuan	679	0	$109 \to j = 51$	Jilin-Fushun	475	0
$47 \to j = 97$	Xi'an-Baotou	899	0	$109 \to j = 108$	Jilin(R)-Changchun(R)	128	41
$47 \to j = 98$	Xi'an-Huhehaote	1064	0	$110 \to j = 47$	Lanzhou-Xi'an	676	0
$47 \to j = 110$	Xi'an-Lanzhou	676	0	$110 \to j = 58$	Lanzhou-Nanchong	1370	0
$47 \to j = 111$	Xi'an-Tianshui	328	0	$110 \to j = 63$	Lanzhou-Bazhong	822	0
$48 \to j = 1$	Shenyang(R)-Beijing(R)	889	5	$110 \to j = 111$	Lanzhou-Tianshui	348	0
$48 \to j = 4$	Shenyang(R)-Tangshan(R)	566	10	$110 \to j = 114$	Lanzhou-Wulumuqi	1892	0
$48 \to j = 50$	Shenyang(R)-Anshan(R)	89	18	$110 \to j = 115$	Lanzhou-Xining	220	0
$48 \to j = 51$	Shenyang-Fushun	61	0	$111 \to j = 47$	Tianshui-Xi'an	328	0
$48 \to j = 99$	Shenyang-Chifeng	574	0	$111 \to j = 110$	Tianshui-Lanzhou	348	0
$48 \to j = 108$	Shenyang(R)-Changchun(R)	303	22	$112 \to j = 57$	Kunming-Chengdu	1100	0
$48 \to j = 109$	Shenyang-Jilin	464	0	$112 \to j = 60$	Kunming-Zigong	913	0
$49 \to j = 50$	Dalian(R)-Anshan(R)	308	18	$112 \to j = 100$	Kunming-Nanning	828	0
$50 \to j = 48$	Anshan(R)-Shenyang(R)	89	18	$112 \to j = 113$	Kunming-Guiyang	638	0
$50 \to j = 49$	Anshan(R)-Dalian(R)	308	18	$113 \to j = 17$	Guiyang-Chongqing	463	0
$51 \to j = 48$	Fushun-Shenyang	61	0	$113 \to j = 61$	Guiyang-Luzhou	447	0
$51 \to j = 109$	Fushun-Jilin	475	0	$113 \to j = 78$	Guiyang-Changsha	949	0
$52 \to j = 53$	Hangzhou(R)-Ningbo(R)	149	25	$113 \to j = 81$	Guiyang-Yongzhou	991	0
$52 \to j = 54$	Hangzhou-Taizhou	323	0	$113 \to j = 82$	Guiyang-Hengyang	1031	0
$52 \to j = 55$	Hangzhou-Wenzhou	449	0	$113 \to j = 100$	Guiyang-Nanning	875	0
$52 \to j = 56$	Hangzhou-Huzhou	81	0	$113 \to j = 104$	Guiyang-Liuzhou	620	0
$52 \to j = 105$	Hangzhou(R)-Nanchang(R)	644	3	$113 \to j = 112$	Guiyang-Kunming	638	0
$52 \to j = 107$	Hangzhou(R)-Yichun(R)	790	1	$114 \to j = 110$	Wulumuqi-Lanzhou	1892	0
$52 \to j = 116$	Hangzhou(R)-Shanghai(R)	202	71	$114 \to j = 115$	Wulumuqi-Xining	2120	0
$53 \to j = 52$	Ningbo(R)-Hangzhou(R)	149	25	$115 \to j = 110$	Xining-Lanzhou	220	0
$53 \to j = 54$	Ningbo(R)-Taizhou(R)	253	22	$115 \to j = 114$	Xining-Wulumuqi	2120	0
$53 \to j = 55$	Ningbo(R)-Wenzhou(R)	376	1	$116 \to j = 18$	Shanghai(R)-Nanjing(R)	301	14
$53 \to j = 105$	Ningbo-Nanchang	778	0	$116 \to j = 19$	Shanghai(R)-Wuxi(R)	126	19
$54 \to j = 52$	Taizhou-Hangzhou	323	0	$116 \to j = 20$	Shanghai(R)-Suzhou(R)	84	101
$54 \to j = 53$	Taizhou(R)-Ningbo(R)	253	22	$116 \to j = 21$	Shanghai(R)-Changzhou(R)	165	3
$54 \to j = 55$	Taizhou(R)-Wenzhou(R)	481	22	$116 \to j = 52$	Shanghai(R)-Hangzhou(R)	202	71
$55 \to j = 52$	Wenzhou-Hangzhou	449	0	$116 \to j = 56$	Shanghai-Huzhou	149	0
$55 \to j = 53$	Wenzhou(R)-Ningbo(R)	376	1	$116 \to j = 86$	Shanghai-Suzhou	575	0
